# “Primary Chronic Granulomatous Sialadenitis of the Parotid Gland: A Rare Case Report”

**DOI:** 10.4317/jced.63394

**Published:** 2026-01-28

**Authors:** Pedro Tapia-Contreras, Pauline Morgan, María José Flores

**Affiliations:** 1 Department of Maxillofacial Surgery, Franco Ravera Zunino Hospital, Chile; 2 Department of Maxillofacial Surgery, Universidad del Desarrollo, Chile; 3 Department of Maxillofacial Surgery, RedSalud Clinic, Vitacura, Chile; 4 Department of Oral Pathology, Franco Ravera Zunino Hospital, Chile; 5 University Andrés Bello, Viña del Mar, Chile

## Abstract

Parotid gland tumors account for 1-3% of head and neck tumors and approximately 70-80% of all salivary gland neoplasms, most of which are benign and predominantly occur in adults. However, non-neoplastic parotid lesions, such as chronic granulomatous sialadenitis, are rare entities with an estimated incidence of 1.7%. They may present as slow-growing, painless masses that are clinically indistinguishable from benign tumors, representing a diagnostic challenge. Case Report: We report the case of a patient with a left parotid mass of two years' evolution, initially evaluated by fine-needle aspiration biopsy (FNAB) (reported as Milan category II, non-neoplastic) and subsequently treated with surgical excision. Histopathological analysis revealed chronic granulomatous sialadenitis, with no evidence of infectious agents or apparent systemic involvement, suggesting a possible autoimmune or idiopathic origin. Discussion: FNAB is the initial diagnostic method of choice due to its low cost, accuracy, and minimal complication rate. It shows a sensitivity of 80%, specificity of 97%, positive predictive value of 90%, and negative predictive value of 94%. However, when cytological findings do not allow confirmation of the nature of the lesion, or when the clinical course is atypical, surgical excision with subsequent histopathological evaluation becomes necessary. Ideally, intraoperative biopsy analysis should be available to establish the definitive diagnosis. Conclusion: Granulomatous reactions in the parotid gland may result from ductal obstruction, specific infections, or systemic granulomatous diseases, making clinico-pathological and immunohistochemical correlation essential. This case highlights the importance of considering inflammatory lesions in the differential diagnosis of parotid masses and the need for a stepwise approach that combines cytology, imaging, and selective surgery.

## Introduction

Primary chronic granulomatous sialadenitis is a rare inflammatory condition of the major salivary glands, characterized by the presence of non-caseating granulomas within the glandular parenchyma. Its etiology is heterogeneous, including infectious causes such as tuberculosis and atypical mycobacteria, autoimmune diseases such as sarcoidosis and Crohn's disease, as well as inflammatory reactions associated with ductal obstruction or necrosis of benign tumors such as Warthin's tumor (1 - 4). Clinically, these lesions may present as firm, painless, slow-growing masses, making their differentiation from primary epithelial neoplasms challenging; histopathological evaluation is therefore indispensable for establishing a definitive diagnosis (5 , 6). The medical literature documents this entity mainly through case reports, reflecting both its rarity and the diagnostic difficulties it entails. Even rarer subtypes, such as xanthogranulomatous or necrobiotic sialadenitis, have been described in association with inflammatory processes secondary to tumor infarction, invasive procedures such as FNAB, or even as a primary presentation without an identifiable cause (3 , 4 , 7 , 8). Moreover, salivary gland involvement in the context of systemic diseases, particularly sarcoidosis, has been reported to clinically and radiologically mimic neoplasms, thereby complicating the preoperative differential diagnosis. In some cases, bilateral involvement of parotid and submandibular glands has been documented, underscoring the need to consider inflammatory conditions in the evaluation of lesions that clinically resemble tumors (2 , 5 , 9). In this context, we present a clinical case that illustrates the clinical, radiological, and histopathological features of this rare entity, along with a review of the relevant literature.

## Case Report

**Figure 1:**
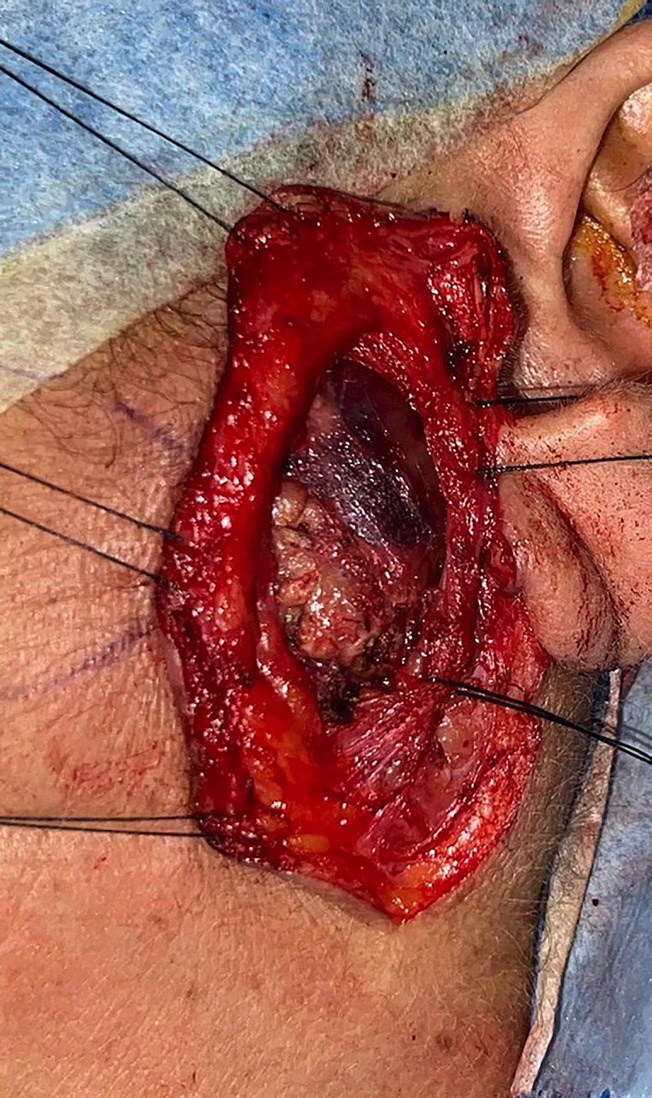
Endaural approach with transparotid dissection to access and excise the lesion.

We report the case of an adult female patient, 60 years old, who presented since 2023 with swelling in the left parotid region. She was evaluated at the maxillofacial department in January 2025, referred with the presumptive diagnosis of a left parotid tumor. The patient reported previous antibiotic treatment with amoxicillin without satisfactory results. Her past medical history was notable for gastritis, managed with omeprazole. She denied the use of anticoagulants or drug allergies. Her surgical history included cholecystectomy, knee arthroscopy, and bladder surgery (for urinary incontinence). Regarding habits, she denied alcohol and tobacco consumption but reported daily heavy use of marijuana. On clinical examination, the patient presented with partial natural dentition and no removable prosthesis. Extraoral examination revealed a firm, well-defined mass of approximately 2 cm in diameter in the left parotid region, without induration or involvement of the overlying skin, which appeared normal. The patient reported pain associated with the lesion, a VAS score of 5 upon palpation and 1 at rest, with an evolution of approximately two years. There was no history of trauma in the area. The clinical features and progression raised suspicion of a tumoral lesion, and surgical management was planned. The patient had previously undergone a FNAB, which was classified as Milan category II and reported as "non-neoplastic." Given the clinical course, surgical excision of the left parotid lesion was performed under general anesthesia in May 2025 (Fig. 1).

1

**Figure 2:**
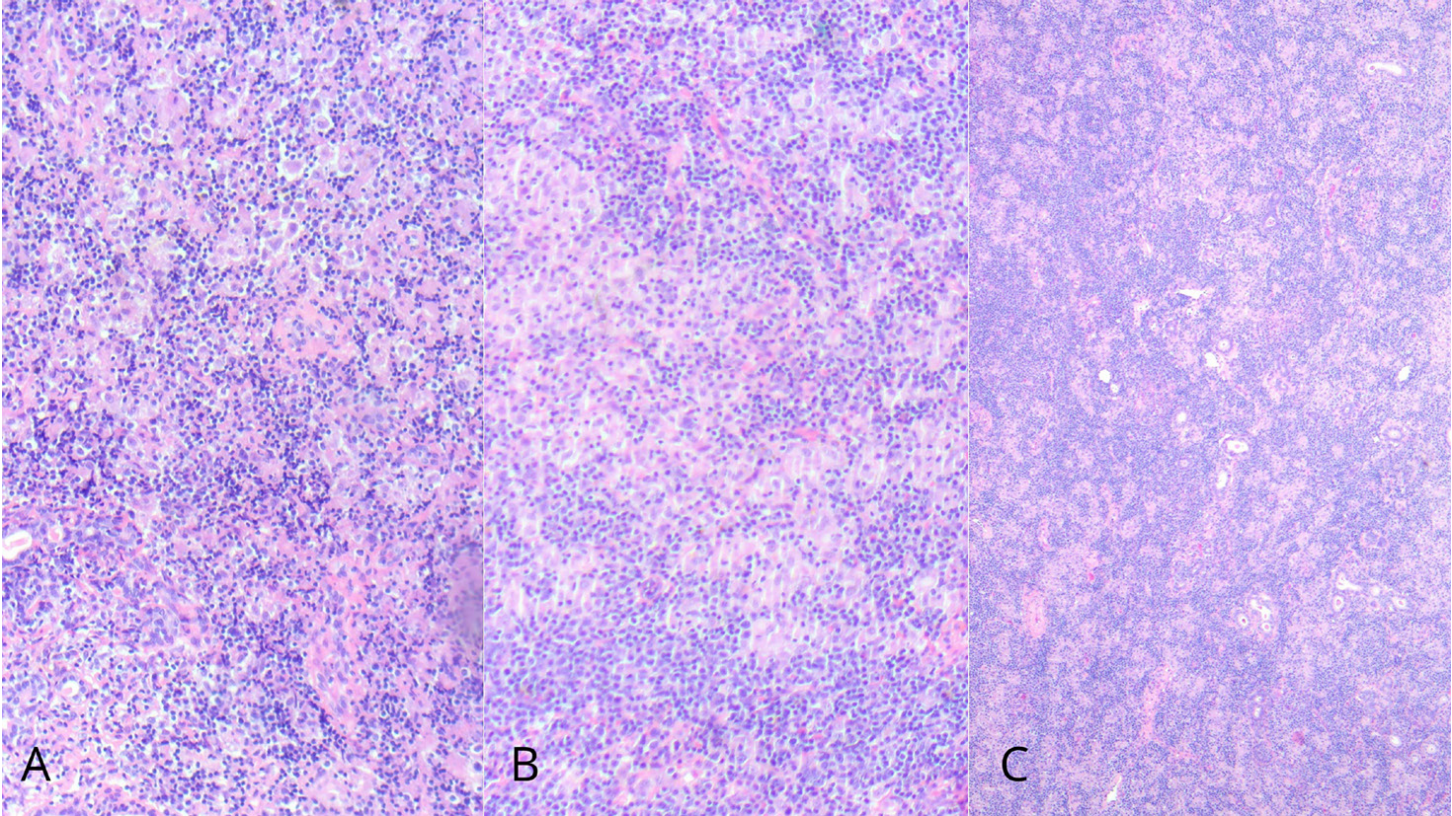
(A–B) Fragments of predominantly serous salivary gland showing a dense lymphoplasmacytic and histiocytic inflammatory infiltrate, with granuloma formation and occasional multinucleated giant cells in areas of complete parenchymal destruction, with preservation of ductal elements. (C) Histiocytic granulomas.

Preoperatively and during hospitalization, including two days postoperatively, she received intravenous cefazolin 1 g every 8 hours and non-steroidal anti-inflammatory drugs (NSAIDs). After hospital discharge, she continued treatment with amoxicillin-clavulanic acid 875/125 mg every 12 hours for 10 days, along with NSAIDs. At the postoperative follow-up one month later, the patient showed satisfactory recovery, with no signs of facial paresis, no salivary fistula, and adequate healing of the surgical site. The skin remained intact, and Stensen's duct was patent with preserved salivary flow. The patient reported improvement in stress levels and was discharged from the maxillofacial surgery team. Histopathological examination of the surgical specimen was conclusive. Microscopic analysis revealed fragments of major serous salivary gland tissue with a dense lymphoplasmacytic and histiocytic inflammatory infiltrate, granuloma formation, and occasional multinucleated giant cells. Extensive destruction of the glandular parenchyma was observed, with partial preservation of ductal structures (Fig. 2).

2

**Figure 3:**
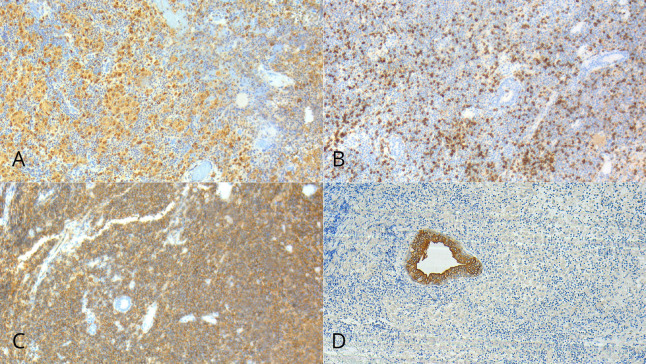
(A) Immunohistochemistry CD68 highlighting histiocytic granulomas; (B) Immunohistochemistry CD3 marking mature T lymphocytes; (C) Immunohistochemistry CD20 marking B lymphocytes; (D) Immunohistochemistry CK AE1/AE3 showing residual salivary gland ducts.

Special stains (Gram, PAS, and Ziehl-Neelsen) were negative for bacteria, fungi, and acid-fast bacilli, respectively. In situ hybridization (CISH) for Epstein-Barr virus was also negative. Immunohistochemical analysis showed positivity for CD3 (T lymphocytes), CD20 (B lymphocytes), CD68 (histiocytes), and CKAE1/AE3 (residual ductal elements) (Fig. 3); markers for Langerhans cell histiocytosis (S100 and CD1a) were negative.

3

Taken together, these findings established the diagnosis of chronic granulomatous sialadenitis of the left parotid gland, with extensive parenchymal destruction and formation of non-necrotizing granulomas. A non-infectious etiology, possibly autoimmune or idiopathic, was considered as the diagnostic hypothesis. Clinico-serological correlation was recommended to rule out systemic entities such as sarcoidosis, Sjögren's syndrome, or other autoimmune diseases. At three months postoperatively, the patient has not received any additional specific pharmacological treatment. She remains under observation, with recommendations for clinical, serological, and imaging follow-up. Prognosis will depend on the local course of the disease and the potential identification of an underlying systemic condition.

## Discussion

Chronic granulomatous sialadenitis is a rare inflammatory condition affecting the major salivary glands, with an estimated incidence of 1.7% in historical series of salivary gland biopsies, and its presentation in the parotid gland is even more uncommon (1). Its clinical relevance lies in the fact that it may present as a slow-growing, firm, well-demarcated mass, mimicking a benign or malignant salivary neoplasm, thereby representing a considerable diagnostic and therapeutic challenge (2 , 3). The most frequent causes of granulomatous salivary inflammation include specific infections such as tuberculosis, syphilis, or deep mycoses; systemic granulomatous diseases such as sarcoidosis and Crohn's disease; and idiopathic or autoimmune conditions such as Sjögren's syndrome (4 , 5). In the present case, the patient had no systemic history nor serological or imaging findings compatible with these entities. Together with the negativity for acid-fast bacilli, fungal elements, Epstein-Barr virus, and histiocytosis markers (CD1a, S100), a possible autoimmune or idiopathic etiology was considered (6 - 8). From a diagnostic standpoint, FNAB was reported as Milan classification category II, non-neoplastic lesion. Although this technique is highly specific and performs well for epithelial lesions, it may be limited in chronic inflammatory processes or in the presence of necrosis, as has been documented in cases of xanthogranulomatous and necrobiotic sialadenitis (2 , 9). When the lesion persists, surgical excision becomes necessary both for establishing the definitive diagnosis and for treatment, as was the case in this patient (10 , 11). Histologically, the characteristic findings of granulomatous sialadenitis include a dense lymphoplasmacytic and histiocytic infiltrate, formation of non-caseating granulomas, and destruction of the glandular parenchyma, often with partial preservation of ductal structures (1 , 6). Previous studies have reported that this pattern may also appear as a secondary reaction to infarcted benign tumors such as Warthin's, or following diagnostic procedures such as aspiration biopsy, triggering an extensive local inflammatory response (2 , 12). However, in the present case, no tumoral elements or necrosis were identified, suggesting a primary process. Regarding therapeutic management, surgery combined with postoperative antibiotic therapy was sufficient to resolve the clinical picture and establish the diagnosis. In the absence of systemic involvement or recurrence, immunosuppressive treatment is not indicated, although clinical and serological follow-up is recommended. Should the lesion recur, alternative strategies have been proposed: new culture and antibiogram with targeted antibiotic therapy, and in refractory cases, a superficial parotidectomy as definitive management (10). In the review of published cases, eight clinical reports and two review articles providing etiological context were identified. Most patients were middle-aged to older adults (range 34-67 years), with a slight female predominance. The most common location was the parotid gland (2 , 9), followed by the submandibular gland (3 , 6), and in one case bilateral involvement of parotid and submandibular glands was reported in the context of systemic sarcoidosis (6). The predominant clinical presentation was a persistent swelling, painful or painless, sometimes accompanied by xerostomia and ocular manifestations. In several reports, imaging showed cystic or solid lesions mimicking neoplasms, and in many cases fine-needle aspiration had been performed prior to surgical excision (2 , 9). The main treatment was gland excision (parotidectomy or submandibulectomy), with favorable outcomes and no recurrences reported in the available follow-up. Only one case showed spontaneous resolution under observation, associated with systemic sarcoidosis. These findings confirm that the evidence comes almost exclusively from isolated case reports (1 , 4 , 7), with generally favorable outcomes after surgical management. The literature available on granulomatous sialadenitis remains extremely limited, almost exclusively restricted to isolated case reports and small series. This lack of prospective studies or cohorts makes it difficult to establish robust conclusions regarding its clinical behavior, etiological factors, or standardized surgical management protocols. Most cases described have presented as glandular lesions initially suspected to be neoplasms, reflecting both the rarity of the entity and the risk of diagnostic confusion. In this scenario, the surgeon faces a significant challenge, as diagnostic confirmation largely depends on the histopathological evaluation of the surgical specimen.

## Conclusions

This case highlights the importance of considering chronic granulomatous sialadenitis in the differential diagnosis of persistent parotid masses. A stepwise approach combining clinical evaluation, cytology, imaging, and histopathology with immunohistochemical support enables an accurate diagnosis and guides appropriate intervention.

## Data Availability

The datasets used and/or analyzed during the current study are available from the corresponding author.
